# Maintenance effects of a multilevel workplace intervention to reduce sedentary time: twenty-four-month follow-up of the group randomized clinical trial *‘Stand and Move at Work’*

**DOI:** 10.1186/s12966-025-01731-w

**Published:** 2025-04-07

**Authors:** Krista S. Leonard, Miranda Larouche, Nathan R. Mitchell, Sarah A. Rydell, Meynard John Toledo, Sarah L. Mullane, Kristina Hasanaj, Junia N. de Brito, Matthew P. Buman, Mark A. Pereira

**Affiliations:** 1 College of Health Solutions, Arizona State University, Phoenix, USA; 2 School of Public Health, Department of Epidemiology & Community Health, University of Minnesota, Minneapolis, USA; 3 Center for Economic and Social Research, University of Southern California, Los Angeles, USA; 4 Johnson and Johnson Health and Wellness Solutions, New Brunswick, USA; 5 Feinberg School of Medicine, Northwestern University, Chicago, IL USA; 6 Medical School, Department of Family Medicine and Community Health, University of Minnesota, Minneapolis, MN USA

**Keywords:** Workplace, Multilevel, Sedentary behavior, Maintenance

## Abstract

**Background:**

The long-term impact of multilevel workplace sedentary behavior interventions has not been established beyond 12-months. We conducted a 2-arm group randomized trial examining the 24-month efficacy of a multilevel workplace intervention with sit-stand workstations (SSW) relative to the same multilevel intervention with delayed SSW implementation until 12-months.

**Methods:**

Worksites (*N* = 24 worksites, *N* = 630 employees) were randomized to participate in *Stand and Move at Work* and received: (a) *STAND* + , a 12-month multilevel behavioral intervention targeting reductions in sedentary time and increases in light physical activity (LPA) with SSW delivery during the 12-months or (b) *MOVE* + , the same *multilevel* intervention, however with SSW delivery at the end of the 12-month primary assessment period. We present maintenance endpoints (24-month follow-up) of objectively measured sedentary behavior variables as well as cardiometabolic biomarkers of the total sample and an at-risk exploratory dysglycemic (prediabetes or diabetes) subgroup per study arm.

**Results:**

All worksites (*N* = 24; from academic [*n* = 8], industry/healthcare [*n* = 8], and government [*n* = 8] sectors) were retained and participated in 24-month follow-up data collection. A total of 464 participants (248 *STAND* + *,* 216 *MOVE* + ; 19 ± 6 per worksite; 45.8 ± 10.6 years of age, 73% female) completed the 24-month assessment. At 24 months, the adjusted within-arm difference in sitting was -37.3 (CI:—51.9, -22.7) min per 8 h workday for *STAND* + and -23.4 (-39.7, -7.0) min per 8 h workday for MOVE + *.* Findings at 12-months were reproduced at 24-months, in which the majority of reductions in sitting translated to increasing standing with minimal change in LPA. There were no significant changes in cardiometabolic risk within the total sample, while there were some significant changes in triglycerides and blood pressure for the dysglycemic participants.

**Conclusions:**

Multilevel workplace interventions incorporating SSWs have the potential to sustain reductions in workplace sedentary time through 24-months. Further, delayed introduction of SSWs following a 12-month multilevel workplace intervention seem to produce similar sitting time reductions relative to immediate introduction. SSWs are a robust environmental stimulus within multilevel interventions targeting workplace sedentary behavior. A larger sample size is needed to detect concomitant impact on cardiometabolic health.

**Trial registration:**

ClinicalTrials.gov Identifier: NCT02566317. Registered on 2 October 2015, the first participant enrolled 11 January 2016. https://clinicaltrials.gov/ct2/show/NCT02566317. See Consort checklist.

**Supplementary Information:**

The online version contains supplementary material available at 10.1186/s12966-025-01731-w.

## Introduction

The rise in desk-based occupations has led to office workers spending upwards of 70–90% of the workday seated [1], increasing the risk for cardiometabolic disease and premature mortality [2–4]. Thus, the workplace has become an opportune environment to target sitting. Research shows that replacing workplace sitting with standing or light-intensity physical activity (LPA) has beneficial implications for cardiometabolic health and may reduce mortality risk [5]. Specifically, several studies have shown that multilevel workplace interventions (i.e., targeting multiple levels of influence such as the individual, physical environment, and organization) coupled with sit-stand workstations (SSW’s) result in reduced workplace sitting time [6–9]. Moreover, multilevel workplace interventions have the potential to produce clinically meaningful changes in cardiometabolic risk factors among “healthy” workers as well as those at risk for prediabetes or diabetes, largely facilitated by behavior change (e.g., reduced sedentary behavior and increased activity) [9, 10]. However, whether these interventions can facilitate sitting reductions and improved cardiometabolic risk factors to be sustained in the long-term remains largely unknown.

Recent group randomized-controlled trials utilizing a multilevel intervention with SSW’s, demonstrated large reductions in workplace sitting time (45–60 min/8 h workday) between 3- and 12-months follow up [9, 11, 12]. The longest current follow-up period conducted by Zhu et al. [13] observed a decrease in workplace sitting time also of approximately 52.6 min/8 h workday at 18-months follow up. However, this study was limited by lack of randomization and a small sample size [14]. Although these trials resulted in promising short-term reductions immediately following the interventions, several reviews of workplace sedentary behavior reduction interventions have been unable to draw conclusions on long-term effects due to the lack of long-term follow-up periods [15–18].

To address this gap, this paper presents findings from the *Stand & Move at Work* (*SMW*) trial [9, 19], a 12-month multilevel workplace sedentary reduction intervention, to determine long-term follow-up maintenance effects at 24-months. In *SMW*, worksites were randomized to receive either, (a) *STAND* + *,* a multilevel intervention implementing SSW’s simultaneously, or (b) *MOVE* + *,* the same multilevel intervention with delayed implementation of SSW’s after completing the 12-month intervention. After a 12-month multilevel workplace intervention, the *STAND* + group exhibited ~ 60 min/8 h workday reduction in workplace sitting, largely replaced with standing, and exhibited favorable changes in cardiometabolic risk score (CMR) within a dysglycemic sub-sample (i.e., diabetes diagnosis or fasting blood glucose ≥ 100 mg/dL) [9].

The purpose of this current study was to examine the long-term maintenance (24-month) impact on workplace sitting and LPA as well as cardiometabolic risk factors following exposure to a 12-month multilevel intervention with SSWs (i.e., *STAND* +) and the impact of delayed SSW implementation following 12 months of exposure to a multilevel intervention (i.e., *MOVE* +).

## Methods

### Participants

Full worksite and employee eligibility criteria and enrollment strategies have been published [19, 20]. In brief, eligible worksites: (a) were small to moderate in size (i.e., 20 – 60 employees), (b) had > 80% of employees working full time (on-site), (c) had predominately seated desk-based office work with < 10% of SSW users, and (d) were not currently participating in a worksite wellness program targeting sitting or increases in physical activity. Worksite leadership must have exhibited willingness to be randomized to either study arm and have SSW installed at the worksite. Worksites were recruited in the Phoenix, AZ and Minneapolis/St. Paul, MN, USA greater metropolitan regions and were selected using purposive sampling across academic, industry/healthcare, and government sectors. All employees employed at eligible worksites were invited to participate and were enrolled if eligible. To participate, employees had to be 18 years or older and in generally good health with the ability to safely reduce sitting and increase standing and LPA. Institutional Review Board approval was given to the Arizona State University and the University of Minnesota study protocol. All participants signed informed consent prior to baseline.

### Study design

*Stand & Move at Work* (*SMW*) was a cluster-two-arm group randomized design in which worksites (*N* = 24) were stratified by workplace sectors (i.e., academic, industry/healthcare, and government) and nested within geographical regions (i.e., Phoenix, AZ and Minneapolis/St. Paul, MN). Study protocol [19] and primary findings [9] have been published elsewhere. A simple randomization procedure was used following stratification among the three sectors (i.e., academic, industry/healthcare, and government) nested within each of the two regions (i.e., Phoenix, AZ and Minneapolis/St. Paul, MN), which was performed by the study biostatistician. Four worksites were enrolled every 2 months between January 2016 and November 2016 to avoid seasonal effects. Workplace activity and cardiometabolic risk factor variables were collected between February and December of 2017 for 12-months, and 24-month (maintenance) outcome data was collected between January and December of 2018.

Worksites were randomized to either *STAND* + , a multilevel intervention plus SSWs, or *MOVE* + , the same multilevel intervention with delayed SSW implementation after the 12-month primary outcome assessment. Prior to any data collection, a pragmatic decision in consultation with National Institutes of Health (NIH), was made to provide *MOVE* + worksites with SSWs immediately following the 12-month assessment, rather than upon completion of the study at 24 months to create balance between the two active interventions arms. That is, both *STAND* + and *MOVE* + worksites received the same 12-month multilevel intervention, however the *MOVE* + worksites did not receive SSWs until after the 12-month intervention was complete (as opposed to *STAND* + worksites receiving SSWs at baseline). Thus, this unique design allows us to examine within-arm changes between 12- and 24-months in response to a 12-month multilevel intervention with SSWs (i.e., *STAND* +) as well as the impact of a 12-month multilevel intervention with delayed SSW implementation starting 12 months (*MOVE* +).

### Interventions

The full description of the multilevel intervention is published elsewhere [21]. The *SMW* interventions were derived from the social ecological model encompassing workplace changes at the organizational/policy (e.g., managerial support), environmental (e.g., signage), social (e.g., contests, role modeling), and individual (e.g., goal setting, education) levels and designed to reduce sitting and increase LPA at work. Worksites were responsible for identifying employee(s) to serve as advocate(s) who were responsible for delivering intervention components and played an active role with study participants. Advocate(s) received training and monthly calls from the research study team and served as the primary contact link between the research study team and worksites. At the start of the study, the *MOVE* + participants were given a goal of obtaining ≥ 30 min of additional LPA over the course of the workday. The *STAND* + participants were given this same LPA goal with an additional goal of increasing standing time to 50% of desk-based worktime. It is important to note that the multilevel intervention was intended as a 12-month intervention; advocates were asked to deliver intervention components to the worksites in both study arms from baseline to 12-months. The 12- to 24-month period was intended as a follow-up period for the STAND + study arm, while in the MOVE + study arm SSWs were distributed and participants were given ergonomic advice on how to use their SSWs.

### Measures

#### Demographic variables

Age, race, sex, education, and job type were assessed via an online survey (Qualtrics, Salt Lake City, UT) at baseline.

#### Workplace sitting time

To assess sitting time at work, the activPAL3c micro accelerometer (PAL Technologies Limited, Glasgow, United Kingdom), a valid measure of posture, was used [22, 23]. To supplement this, a log was sent to participants to determine daily work and nonwork time as well as wake and sleep time. Participants were asked to wear the waterproofed device (i.e., can wear while bathing/water-activities) for seven consecutive days on their right-thigh. Any long bouts of continuous sedentary or standing time exceeding 6 h were classified as non-wear time and excluded from analyses. Any wake periods with ≤ 10 h of wear time or work periods with < 4 h of wear time were also excluded. Sleep logs provided time in bed, though an automated algorithm was used when not available [24]. Sensitivity analyses revealed no difference in estimates when sleep logs vs automated algorithm methods were used. All outcomes are provided as work periods (as the intervention was specific to the workplace) and as total wake time to assess possible compensation effects outside of work. All work periods were standardized to an 8 h workday (i.e., standardized minutes = observed minutes × 480/observed minutes of wear time), whereas total wake times were standardized to a 16 h day. Time spent in LPA and moderate-to-vigorous physical activity (MVPA) were derived from step counts as < 100 steps/minute for LPA and ≥ 100 steps/minute for MVPA [25]. In total, the following outcomes are derived for both work periods and total waking time: sitting (min/day); standing (min/day); LPA (min/day); MVPA (min/day); total physical activity, combined LPA and MVPA (min/day); sit-to-stand transitions (number of transitions/h of sitting); and sitting time accrued in bouts ≥ 30 m (min/day). Participants in both study arms received standardized reports of their activPAL data.

#### Cardiometabolic risk biomarkers

Measurements of body weight, and resting blood pressure, as well as fasting and venous serum concentration of glucose, insulin, triglycerides, and LDL- and HDL-cholesterol were assessed following procedures from the original trial with batch processing [9, 19]. Cardiometabolic risk score (CMR) and individual biomarker scores were also examined in a continuous fashion [26], also following procedures from the original trial [9, 19]. An exploratory subgroup of dysglycemic patients (i.e., diagnosed with diabetes or a fasting blood glucose ≥ 100 mg/dL) was also examined at 24-months, within each study arm.

### Sample size and statistical analysis

Analyses were performed in SAS 9.4 (SAS Institute Cary, N.C.). To remain consistent with reporting of our primary outcomes [9], intent to treat procedures without imputation were followed at the level of the worksite (the unit of randomization), with 24 worksites being randomized and analyzed. Individuals within worksite were included when 24-month data were available. The senior statistician was blinded to group assignment and the analyst/programmer was blinded until the statistical models were finalized. Individuals who became pregnant or lactating during the trial were excluded from cardiometabolic analyses. Each outcome was defined as 24-month change from baseline and 24-month change from 12 months. Distributions were examined and plotted against baseline to identify implausible values and influential points prior to analysis. Sensitivity analyses using log transformed and winsorized (3rd quartile + 1.5*SD) outcomes gave similar results and are not reported. Effects for *STAND* + and *MOVE* + were tested separately. Linear mixed models were used to examine within group changes. The group randomized design was accounted for using a random effect for site nested within treatment. Models were adjusted for baseline values of the respective outcome and a priori selected covariates: age, sex, race/ethnicity, and baseline BMI.

## Results

**Fig. 1:**
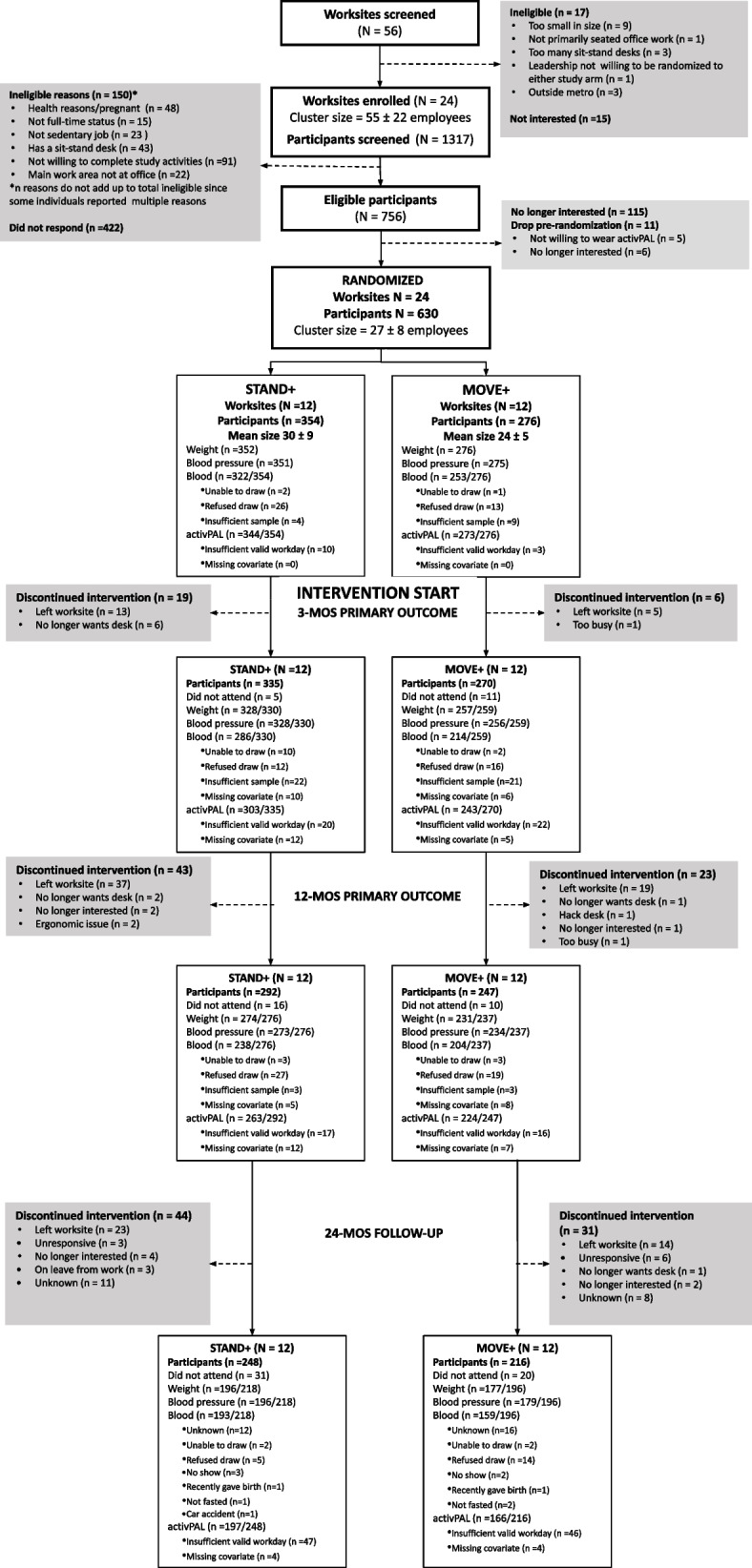
Worksite and participant flow

All worksites (*N* = 24) were retained (12 per study arm) in each region/sector stratum through 24-months. Figure 1 presents an updated CONSORT diagram to the original trial extending retention through 24-months. A total of 464 participants (248 *STAND* + *,* 216 *MOVE* + ; 19 ± 6 per worksite; 45.8 ± 10.6 years of age, 73% female) were retained from baseline for the 24-month follow up with an overall 24-month participant retention rate of 74%. At 24-months the retained sample size per worksite was 21 ± 7 participants (range:10–35) for *STAND* + , and 18 ± 5 (range:10–26) for *MOVE* + . Loss-to-follow-up from baseline through 12-months has been published elsewhere [9]. Between 12- and 24- months, *STAND* + lost 44 participants and *MOVE* + lost 31 participants. Table 1 describes the baseline characteristics of the 24-month sample in total and by study arm. Supplementary Table 1 provides a comparison of the demographics and baseline activity and cardiometabolic risk biomarkers of the baseline and 12- and 24-month analytical samples by study arm. Sample retention was comparable between study arms and demographic, baseline activity, and cardiometabolic risk characteristics of participants retained and those lost-to-follow-up from 12-months to 24-months were similar.

**Table 1:** Demographics of the 24-month sample

	*STAND* +				*MOVE* +				Total (*STAND* + and *MOVE* +)			
	All participants		Dysglycemic subgroup		All participants		Dysglycemic subgroup		All participants		Dysglycemic subgroup	
	n	(%)	n	(%)	n	(%)	n	(%)	n	(%)	n	(%)
N worksites	12	(50.0)	–	–	12	(50.0)	–	–	24	(100.0)	–	–
N individuals	248	(53.4)	74	(29.8)	216	(46.6)	47	(21.8)	464	(100.0)	121	(26.1)
Region												
Phoenix, Arizona	137	(55.2)	45	(60.8)	106	(49.1)	27	(57.5)	243	(52.4)	72	(59.5)
Minneapolis/St. Paul, Minnesota	111	(44.8)	29	(39.2)	110	(50.9)	20	(42.6)	221	(47.6)	49	(40.5)
BMI (M ± SD)	30.2 ± 7.6		33.6 ± 8.6		29.1 ± 6.5		32.3 ± 7.0		29.7 ± 7.2		33.1 ± 8.0	
Age (years) (M ± SD)	46.9 ± 10.7		50.6 ± 10.0		44.7 ± 10.4		48.4 ± 10.3		45.8 ± 10.6		49.7 ± 10.2	
Race												
Non-Hispanic White	168	(67.7)	52	(70.3)	158	(73.1)	31	(66.0)	326	(70.3)	83	(68.6)
Hispanic	35	(14.1)	7	(9.5)	25	(11.6)	5	(10.6)	60	(12.9)	12	(9.9)
Non-Hispanic Black	18	(7.3)	7	(9.5)	4	(1.9)	1	(2.1)	22	(4.7)	8	(6.6)
Non-Hispanic Asian	10	(4.0)	2	(2.7)	15	(6.9)	2	(4.3)	25	(5.4)	4	(3.3)
Other/Multiracial/Unknown	17	(6.9)	6	(8.1)	14	(6.5)	8	(17.0)	31	(6.7)	14	(11.6)
Female	206	(83.1)	55	(74.3)	133	(61.6)	27	(57.5)	339	(73.1)	82	(67.7)
Education												
Less than college	8	(3.2)	2	(2.7)	14	(6.5)	6	(12.8)	22	(4.7)	8	(6.6)
College/Some college	169	(68.1)	52	(70.3)	120	(55.6)	22	(46.8)	289	(62.3)	74	(61.2)
Graduate/Professional	62	(25.0)	16	(21.6)	75	(34.7)	15	(31.9)	137	(29.5)	31	(25.6)
Unknown	9	(3.6)	4	(5.4)	7	(3.2)	4	(8.5)	16	(3.4)	8	(6.6)
Work sector												
Academic	86	(34.7)	25	(33.8)	70	(32.4)	11	(23.4)	156	(33.6)	36	(29.8)
Industry/healthcare	74	(29.8)	23	(31.1)	64	(29.6)	21	(44.7)	138	(29.7)	44	(33.4)
Government	88	(35.5)	26	(35.1)	82	(38.0)	15	(31.9)	170	(36.6)	41	(33.9)
Job type												
Executive	31	(12.5)	9	(12.2)	32	(14.8)	6	(12.8)	63	(13.6)	15	(12.4)
Professional	130	(52.4)	40	(54.1)	124	(57.4)	21	(44.7)	254	(54.7)	61	(50.4)
Clerical	82	(33.1)	23	(31.1)	54	(25.0)	16	(34.0)	136	(29.3)	39	(32.2)
Unknown	5	(2.0)	2	(2.7)	6	(2.8)	4	(8.5)	11	(2.4)	6	(5.0)

*BMI* body mass index

At 24-months, 197 *STAND* + and 166 *MOVE* + participants completed the activPAL assessment. Overall, activPAL wear time during waking hours was high: 84% of *STAND* + participants had ≥ 5 valid days and 3 valid work periods (6.5 ± 1.5 valid days and 4.0 ± 1.1 work periods) with a waking wear time of 15.2 ± 1.0 h per valid day and 8.4 ± 1.0 h per valid work period. Similarly, 89% of *MOVE* + participants had ≥ 5 valid days and 3 valid work periods (6.7 ± 1.2 valid days and 4.1 ± 1.0 work periods), with waking wear time of 15.2 ± 0.8 h per valid day and 8.3 ± 0.8 h per valid work period.

**Fig. 2:**
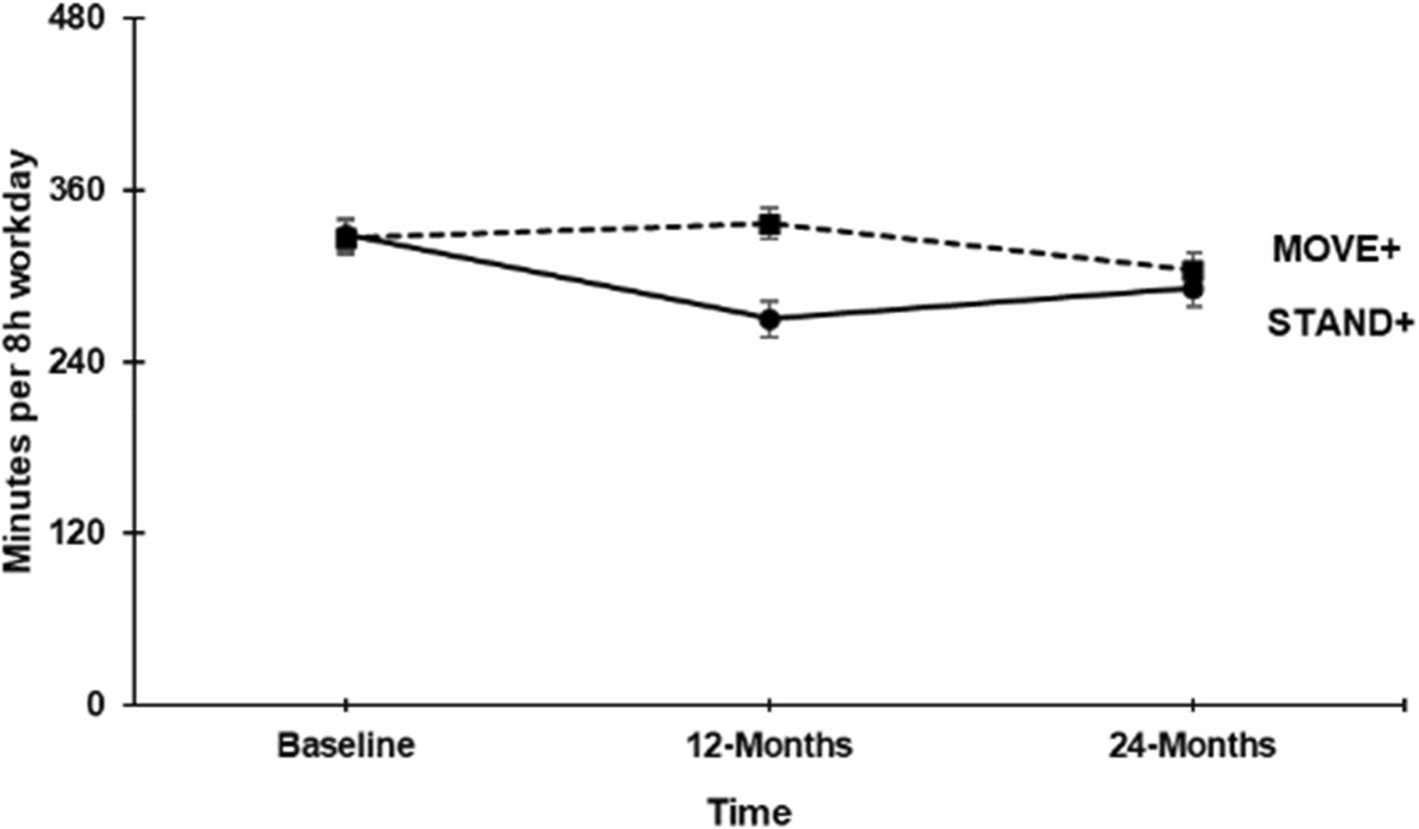
Mean workplace sitting time in STAND + and MOVE + study arms at baseline, 12- and 24-months. Note. Circle data points refer to the *STAND* + study arm; Square data points refer to *MOVE* + study arms; Error bars are 95% confidence intervals

The results of the 24-month maintenance outcomes of activPAL-measured variables for work time (standardized to an 8 h workday; Fig. 2) and total waking time (standardized to a 16 h day), are displayed in Table 2. For *STAND* + , the adjusted within-arm mean difference in sitting time was 21.9 (9.3, 34.5) mins per 8 h workday at 24 months relative to 12 months, and -37.3 (-51.9, -22.7) mins per 8 h workday relative to baseline. For *MOVE* + , the adjusted within-arm mean difference in sitting time was -23.4 (-39.7, -7) mins per 8 h workday at 24 months relative to 12 months, and -31.8 (-43.5, -20.2) mins per 8 h workday relative to baseline. The adjusted within-arm mean difference in standing time for *STAND* + was -18.8 (-30.7, -7.02) mins per 8 h workday at 24 months relative to 12 months and 37.2 (21.8, 52.6) mins per 8 h workday relative to baseline. The adjusted within-arm mean difference in standing time in *MOVE* + was 32.8 (-43.5, -20.2) mins per 8 h workday at 24 months relative to 12 months and 24.4 (6.8, 41.9) mins per 8 h workday relative to baseline. Both study arms observed reductions in sitting largely translated into standing behaviors and changes in LPA and MVPA activity were minimal.

**Table 2:** Intervention effects on objectively measured work time and total time activity variables, by study arm in minutes, at 0, 12, and 24 months of the 24 month analytic sample

		*Baseline*	*12-month*	*24-month*	*0–24 month*, Difference (95% CI)	*12–24 month*, Difference (95% CI)
		Mean ± SD	Mean ± SD	Mean ± SD		
***STAND* + *(n* = *197)***					Long term effects	Maintenance Post 12-Month Intervention
	Work periods					
	Sitting	328.6 ± 81.8	269.8 ± 87.4	291.2 ± 91.8	-37.3 (-51.91, -22.7)	21.9 (9.3, 34.5)
	Standing	114.2 ± 77.7	169.8 ± 84.9	151.3 ± 88.5	37.2 (21.8, 52.6)	-18.8 (-30.7, -7.02)
	LPA	31.5 ± 15.7	33.6 ± 16.5	31.5 ± 17.1	0.0 (-2.0, 2.1)	-2.2 (-4.3, -0.1)
	MVPA	5.7 ± 4.6	6.8 ± 6.5	6.1 ± 4.7	0.3 (-0.3, 0.8)	-0.9 (-1.7, -0.1)
	LPA + MVPA	37.2 ± 17.2	40.4 ± 18.9	37.6 ± 18.6	0.3 (-1.9, 2.5)	-3.1 (-5.3, -0.8)
	Prolonged sitting (> 30 min)	142.2 ± 91.3	110.5 ± 77.7	129.2 ± 91.1	-12.4 (-25.5, 0.7)	20.0 (7.6, 32.3)
	Sit-stand transitions^a^	7.0 ± 4.4	8.3 ± 6.4	7.6 ± 6.1	0.6 (-0.3, 1.4)	-0.7 (-1.5, 0.2)
	Total time					
	Sitting	611.8 ± 100.0	567.8 ± 113.4	596.2 ± 110.1	-15.0 (-29.1, -1.0)	28.9 (13.8, 43.9)
	Standing	248.1 ± 86.3	291.3 ± 98.5	269.3 ± 97.8	21.1 (7.97, 34.3)	-22.1 (-35.8, -8.5)
	LPA	82.5 ± 27.1	82.5 ± 28.8	77.2 ± 28.7	-5.5 (-11.6, 0.6)	-5.5 (-8.7, -2.3)
	MVPA	17.7 ± 8.0	18.4 ± 8.6	17.3 ± 7.3	-0.5 (-1.6, 0.5)	-1.3 (-2.2, -0.3)
	LPA + MVPA	100.2 ± 31.8	101.0 ± 33.7	94.5 ± 32.3	-6.0 (-12.9, 0.9)	-6.7 (-10.3, -3.1)
	Prolonged sitting (> 30 min)	302.1 ± 106.9	275.9 ± 109.6	305.1 ± 110.5	3.4 (-11.5, 18.4)	30.2 (14.7, 45.7)
	Sit-stand transitions^a^	5.9 ± 2.1	6.2 ± 2.2	5.8 ± 2.1	-0.1 (-0.5, 0.2)	-0.4 (-0.7, -0.1)
***MOVE* + *(n* = *166)***					Long term effects	Maintenance Post 12-Month Intervention
	Work periods					
	Sitting	326.5 ± 77.2	336.6 ± 71.6	304.1 ± 76.5	-23.4 (-39.7, -7)	-31.8 (-43.5, -20.2)
	Standing	114.0 ± 75.5	104.8 ± 70.4	137.7 ± 75.8	24.4 (6.8, 41.9)	32.1 (21.4, 42.8)
	LPA	32.7 ± 16.0	32.2 ± 14.8	31.5 ± 14.2	-0.9 (-3.5, 1.7)	-0.8 (-2.9, 1.4)
	MVPA	6.8 ± 5.5	6.4 ± 5.5	6.7 ± 5.0	-0.1 (-0.7, 0.5)	0.5 ('-0.2, 1.0)
	LPA + MVPA	39.5 ± 17.5	38.6 ± 16.3	38.2 ± 15.6	-1.0 (-3.6, 1.5)	-0.2 (-2.9, 2.5)
	Prolonged sitting (> 30 min)	144.6 ± 83.4	164.4 ± 87.9	152.4 ± 83.5	7.5 (-8.8, 23.9)	-11.4 (-23.0, 0.3)
	Sit-stand transitions^a^	6.5 ± 3.9	5.5 ± 1.9	5.6 ± 3.2	0.2 (-2.2, 2.6)	-4.0 (-12.3, 4.3)
	Total time					
	Sitting	603.0 ± 89.2	618.6 ± 82.9	591.2 ± 90.5	-11.6 (-27.9, 4.8)	-26.4 (-38.3, 14.6)
	Standing	251.9 ± 78.5	240.6 ± 71.9	266.4 ± 78.4	14.2 (-2.5, 30.8)	24.7 (14.5, 35.0)
	LPA	85.9 ± 30.2	82.1 ± 27.8	83.4 ± 29.1	-2.5 (-5.8, 0.8)	1.4 (-2.0, 4.8)
	MVPA	19.2 ± 7.2	18.7 ± 7.9	18.9 ± 7.3	-0.2 (-1.0, 0.7)	0.3 (-0.7, 1.3)
	LPA + MVPA	105.1 ± 33.5	100.8 ± 31.5	102.4 ± 33.2	-2.7 (-6.4, 1.0)	1.7 (-2.0, 5.4)
	Prolonged sitting (> 30 min)	298.4 ± 98.4	319.6 ± 107.0	311.6 ± 100.6	12.8 (-2.7, 28.3)	-7.9 (-21.6, 5.8)
	Sit-stand transitions^a^	5.9 ± 1.9	5.5 ± 1.9	5.5 ± 1.8	-0.3 (-0.6, -0.1)	0.1 (-0.2, 0.2)

Linear mixed models were used to analyze the change in outcomes, accounting for age, sex, and race, and BMI. Random effect for site nested within treatment group

*LPA* Light-intensity physical activity, *MVPA* Moderate-vigorous physical activity. Work period outcomes have been standardized to an 8 h workday (minutes)

^a^Sit-stand transitions are expressed as number of transitions per sedentary hour

CMR score data were available for 175 and 150 *STAND* + and *MOVE* + participants, respectively. The individual components of CMR along with additional anthropometric and chronic disease values of the total and dysglycemic subgroups by study arm are presented in Table 3.. For both the total and dysglycemic subgroup samples for *STAND* + and *MOVE* + , effects were mostly small. However, in the dysglycemic subsample there was a significant reduction in triglycerides for STAND + from 12 to 24 months, and for MOVE + from 0 to 24 months there were reductions in triglycerides and diastolic blood pressure.

**Table 3:** Intervention effects on cardiometabolic risk biomarkers of the 24-month analytical sample by study arm

		Baseline	12-month	24-month	Difference, 0-24 months (95% CI)	Difference, 12-24 months (95% CI)
		M±SD	M±SD	M±SD		
***STAND*+**						
* Total (n=175)*						
	CMR (sum of Z scores)	0.0±0.7	0.0±0.7	0.0±0.7	-0.0 (-0.1, 0.0)	0.0 (-0.0, 0.1)
	Fasting glucose (mg/dL)	98.0±41.7	96.6±30.6	96.3±32.2	-1.9 (-4.8, 0.9)	-0.5 (-3.9, 2.8)
	Fasting insulin (uU/mL)	76.1±58.9	71.3±62.4	83.3±104.4	1.9 (-4.8, 8.6)	5.6 (-5.8, 17.0)
	HDL-cholesterol (mg/dL)	60.6±17.3	61.3±17.8	59.4±16.9	0.8 (-3.1, 1.6)	-1.6 (-3.5, 0.3)
	Triglycerides (mg/dL)	116.2±61.1	117.6±60.7	110.0±61.8	-7.5 (-17.1, 2.0)	-7.8 (-16.4, 1.3)
	Diastolic BP (mm Hg)	77.4±10.1	77.6±10.0	76.8±9.6	-0.6 (-1.8, 0.5)	-1.0 (-2.2, 0.3)
	Systolic BP (mm Hg)	124.7±15.1	122.5±15.1	122.7±14.8	-2.0 (-4.1, 0.2)	0.1 (-2.1, 2.3)
	Additional outcomes					
	LDL-cholesterol (mg/dL)	109.5±29.6	110.0±30.9	110.9±29.6	0.2 (-3.1, 3.5)	0.3 (-3.5, 4.2)
* Dysglycemic subgroup (n=43)*						
	CMR (sum of Z scores)	0.7±0.8	0.5±0.7	0.5±0.7	-0.2 (-0.3, 0.0)	0.03 (-0.1, 0.2)
	Fasting glucose (mg/dL)	132.7±72.6	123.7±50.4	123.2±53.6	-9.7 (-19.6, 0.12)	-0.8 (-13.6, 12.1)
	Fasting insulin (uU/mL)	122±80.8	102±100.0	123.8±97.3	2.3 (-19.8, 24.4)	20.4 (-19.6, 60.5)
	HDL-cholesterol (mg/dL)	54±14.2	55±15.2	54.1±16.0	0.4 (-3.6, 4.4)	-0.3 (-2.7, 2.2)
	Triglycerides (mg/dL)	134.3±63.3	132.6±66.0	122.2±68.2	12.8 (-27.9, 2.3)	-11.5 (-22.9, -0.0)
	Diastolic BP (mm Hg)	83.3±10.3	82.2±10.0	80.7±9.5	-3.1 (-6.3, 0.1)	-2.1 (-5.4, 1.3)
	Systolic BP (mm Hg)	136.5±15.5	131.5±15.7	132.6±15.5	-4.5 (-10.9, 1.9)	0.6 (-4.6, 5.9)
	Additional outcomes					
	HbA1c (%)	6.3±1.5	6.3±1.6			
	LDL-cholesterol (mg/dL)	117.9±10.5	110.5±33.4	111.5±34.5	-8.3 (-16.0, -0.7)	-0.7 (-9.9, 8.6)
	Weight (kg)	99.1±24.6	96.9±25.0	98.2±25.3	-0.7 (-4.4, 2.9)	1.4 (-0.2, 3.0)
	BMI (kg/m2)	35.3±9.1	34.5±9.0	35±9.1	-0.3 (-1.7, 1.0)	0.5 (-0.1, 1.0)
	Total body fat (%)	41.5±10.3	41±10.5	40.2±10.2	-1.3 (-4, 1.5)	-0.4 (-3.0, 2.0)
***MOVE*+**						
* Total (n=149)*						
	CMR (sum of Z scores)	0.0±0.6	0.0±0.6	0.0±0.6	-0.0 (-0.1, 0.0)	-0.0 (-0.1, 0.0)
	Fasting glucose (mg/dL)	92.0±15.3	93.0±15.5	92.8±18.5	0.8 (-1.1, 2.6)	-0.3 (-2.0, 1.5)
	Fasting insulin (uU/mL)	70.2±50.3	64.3±47.0	73.2±56.5	3.5 (-2.9, 10.0)	9.5 (3.5, 15.5)
	HDL-cholesterol (mg/dL)	56.9±17.1	56.7±16.4	56.3±16.6	-0.6 (-2.4, 1.2)	-0.4 (-1.9, 1.1)
	Triglycerides (mg/dL)	128.2±73.8	127.2±68.3	119.6±68.4	-8.2 (-17.4, 1.1)	-7.6 (16.4, 1.3)
	Diastolic BP (mm Hg)	79.2±11.1	77.8±11.3	76.8±10.3	-2.3 (-3.4, -1.2)	-0.9 (-2.5, 0.7)
	Systolic BP (mm Hg)	126.4±16.5	123.7±17.1	122.5±14.8	-3.6 (-5.7, -1.6)	-1.2 (-3.1, 0.8)
	Additional outcomes					
	LDL-cholesterol (mg/dL)	112.27±30.3	115.5±31.1	113.8±29.7	1.5 (-1.7, 4.8)	-1.8 (-5.0, 1.4)
* Dysglycemic subgroup (n=27)*						
	CMR (sum of Z scores)	0.5±0.8	0.38±0.6	0.4±0.7	-0.1 (-0.3, 0.0)	-0.0 (-0.1, 0.1)
	Fasting glucose (mg/dL)	114.7±21.7	113.6±24.4	115.7±30.6	1.0 (-5.1, 7.0)	2.1 (-2.5, 6.7)
	Fasting insulin (uU/mL)	104.2±74.0	86.1±64.4	98.1±82.0	-3.7 (-26.7, 19.3)	13.6 (-4.6, 31.8)
	HDL-cholesterol (mg/dL)	54±20.6	53.2±18.0	54.8±22.9	0.8 (-3.3, 4.8)	1.6 (-2.6, 5.8)
	Triglycerides (mg/dL)	156.2±104.9	140.2±61.7	134.9±62.6	-21.2 (-42.1, -0.4)	-5.3 (-16.8, 6.2)
	Diastolic BP (mm Hg)	82.5±12.4	81.9±12.7	79±10.4	-3.5 (-6.5, -0.5)	-3.0 (-6.2, 0.3)
	Systolic BP (mm Hg)	132.8±20.5	131.5±23.3	128.2±16.2	-3.8 (-10.3, 2.6)	-3.2 (-7.7, 1.2)
	Additional outcomes					
	HbA1c (%)	5.8±0.8	5.9±0.9			
	LDL-cholesterol (mg/dL)	111.3±33.2	115.4±36.9	111.3±37.0	0.9 (-9.5, 11.3)	-5.0 (-11.9, 1.9)
	Weight (kg)	94.7±24.9	96.1±25.8	96.4±27.3	1.7 (-1.1, 4.4)	0.3 (-1.3, 2.0)
	BMI (kg/m2)	32.4±7.8	32.9±7.9	33±8.5	0.6 (-0.4, 1.5)	0.1 (-0.4, 0.7)
	Total body fat (%)	36.8±10.3	37.2±10.0	37.1±10.7	0.3 (-1.4, 2.0)	0.6 (-1.3, 1.3)

Linear mixed models were used to analyze the change in outcomes, accounting for age, sex, and race, and BMI. Random effect for site nested within treatment group. *CMR* summary continuous metabolic risk score, *HDL* high-density lipoprotein, *BP* blood pressure, *BMI* body mass index

## Discussion

The purpose of this study was to examine the long-term maintenance (24-month) of workplace sitting and LPA as well as cardiometabolic risk factors following immediate and delayed implementation of SSW combined with a 12-month multilevel intervention. Findings provide support to the efficacy of multilevel interventions encompassing SSW’s (i.e., *STAND* +) on maintaining workplace sitting time reductions through 24-months – the longest current follow-up period for a workplace intervention. In addition, we found evidence for reducing workplace sitting time by implementing SSW’s following a 12-month multilevel intervention (i.e., *MOVE* +). Overall, our findings provide evidence for the ability of multilevel interventions coupled with SSWs to produce the most robust and sustained reductions in workplace sitting time and lay the groundwork for understanding long-term public health impacts of multilevel workplace sedentary reduction interventions.

Reductions in workplace sitting observed within *STAND* + at 12 months [9] were largely maintained at 24 months. Specifically, although workplace sitting slightly increased from 12 to 24 months by approximately 22 min per 8 h workday, participants maintained an overall almost 40 min per 8 h workday reduction at 24 months compared to baseline. Past research examining long-term effects of non-occupational sedentary behavior reductions have resulted in mixed findings [13, 27–30]. Our findings are similar to Thomsen et al. who found an individually tailored behavioral intervention targeting reductions in daily sitting time resulted in sustained sitting reduction of over 60 min/day at 22-months follow-up [31]. However, our findings are in contrast to studies that found non-occupational sedentary time reductions were not sustained in the long-term [32–34]. Although a shorter follow-up time period, our findings are in line with Zhu et al. [13] who found a 53 min/8 h workday reduction in workday sitting time following the use of sit-stand workstations at 18-month follow-up.

While *MOVE* + exhibited negligible within-group reductions in workplace sitting time from baseline to 12-months [9], workplace sitting time was reduced by over 30 min per 8 h workday at 24-months compared to baseline. This reduction was largely accumulated from 12- to 24-months, after receiving SSW’s following the 12-month primary assessment. Although exposure to the multilevel *SMW* program for 12 months with worksite policy, environment, cultural, social, and individual level strategies may have helped set the foundation for behavioral support, the *MOVE* + study arm likely produced reductions in sitting at 24-months from the addition of the SSW’s on top of consistent program exposure over the first 12-months of the study. However, it is important to note that past studies have found that the maximal benefits of SSWs may not be reached without an accompanying multilevel intervention. For example, Neuhaus and colleagues found that reduction in workplace sitting time were more than doubled in an intervention group with SSWs plus a multilevel intervention compared to an intervention group with SSWs alone [35]. Nevertheless, SSWs provide participants with additional capability and opportunity to reduce sitting behaviors with minimal impact on work productivity and engagement.

Consistent with findings from the first trial [9], the reductions in workplace sitting were largely translated into standing behavior for both *STAND* + and *MOVE* + ; and small effects were seen in physical activity at 24 months. Based on average changes in sitting, standing, and physical activity time, the changes did not meet the overall behavioral targets (e.g.,, 50% increase in standing time). However, the reductions in sitting time during the 24-month follow-up period are clinically meaningful with the potential to improve health outcomes [36]. Findings continue to exhibit negligible compensation for sitting outside of work. Nevertheless, our findings highlight that workplace sitting time reductions are sustained following a 12-month multilevel intervention coupled with SSWs, as well delayed introduction of SSWs following a 12-month multilevel intervention. Past research has shown that use of SSWs tend to decrease over long periods of time [37], however, our findings suggest that worksites may require initial behavioral support to sustain SSW usage for work-related activities.

Minimal effects were seen in cardiometabolic risk factors from baseline to 24-months and 12- to 24-months within the total sample, while there appeared to be some significant effects for triglycerides and blood pressure for the small dysglycemic subgroup for both *STAND* + and *MOVE* + . Experimental research indicates that replacing sitting with standing and/or LPA has the potential to beneficially modify insulin sensitivity and glucose disposal through activation of the large lower-body muscle groups [38–43]. Frequent changes in posture through the use of SSWs may have additional peripheral vascular benefits favorable to cardiometabolic risk [44–46]. As sedentary behaviors independently predicts the risk of future CVD and all-cause mortality [47, 48], there is a need to further explore potential benefits of workplace sedentary behavior interventions on improving cardiometabolic health.

The current study is novel because of the long-term follow-up of device-based workplace sitting time, LPA, and cardiometabolic risk factors following a multilevel, sedentary behavior focused intervention. This study is also among the first to examine the impact of adding SSWs to worksites after exposure to a multilevel behavioral intervention for 12 months. Additional strengths of this study include the generalizability of the results with 24 worksites recruited across three sectors and two states with the longest current follow-up period of 24 months and the use of objective assessments for workplace sitting time and LPA. Some limitations worth noting include the lack of a non-intervention control group, limiting our ability to compare our results to worksites with no intervention. Also, this trial only included full-time sedentary workers of relatively good health with no contraindications to reduce sitting and increase standing and LPA. In addition, because of the differences between the *STAND* + and *MOVE* + intervention groups from 12- to 24-months (i.e., delayed intervention), we were unable to examine between group differences in sustained workplace sitting time and LPA and cardiometabolic outcomes. Finally, although we obtained maintenance data of intervention strategies throughout the 12-month intervention period (e.g., what intervention components were implemented and maintained) [49], we did not collect this data during the 24-month follow-up period. Thus, we are unable to examine what intervention strategies were maintained and/or built upon during the 24-month follow-up.

## Conclusion

In summary, we found that reductions in workplace sitting were largely sustained through 24-month follow-up. Examination of long-term follow-up is critical to determine if the reductions observed in workplace sedentary behaviors, as well as any uptake in standing or moving, has been sustained. Identifying strategies for sustained workplace sedentary behavior reductions in the long-term is essential as this may have positive health outcome implications. The results from the *STAND* + group indicate that even with minimal researcher involvement, multilevel workplace programs coupled with SSWs have the potential to sustain reductions in workplace sitting over 24 months. Further, the delayed introduction of SSWs in the MOVE + study arm allowed us to examine whether delivering a 12-month multilevel intervention followed by the introduction of SSWs would yield similar results to immediate introduction of SSWs. We found similar reduction in sitting time from 12–24 months in the MOVE + arm that we observed in the STAND + arm from 0–12 months, which in one sense replicated our original study results but also suggested that delayed implementation of SSW may have similar impact as immediate implementation. More research is needed to determine potential long-term health implications in response to a multilevel behavioral intervention with SSWs.

## Supplementary Information

Supplementary Material 1.

Supplementary Material 2.

## Data Availability

The datasets supporting the conclusions of this article are available upon request though the co-corresponding authors, Drs. Matthew Buman and Mark Pereira.

## References

[1] Bailey DP. Sedentary behaviour in the workplace: prevalence, health implications and interventions. Br Med Bull. 2021;137(1):42–50. 10.1093/bmb/ldaa039. Cited 2024 Dec 4. 33710270 10.1093/bmb/ldaa039

[2] Zeigler ZS, Mullane SL, Crespo NC, Buman MP, Gaesser GA. Effects of standing and light-intensity activity on ambulatory blood pressure. Med Sci Sports Exerc. 2016;48(2):175–81. 26285021 10.1249/MSS.0000000000000754

[3] Gao W, Sanna M, Chen YH, Tsai MK, Wen CP. Occupational sitting time, leisure physical activity, and all-cause and cardiovascular disease mortality. JAMA Netw Open. 2024;7(1):e2350680–e2350680. Available from: https://jamanetwork.com/journals/jamanetworkopen/fullarticle/2814094. Cited 2024 Dec 4. 38241049 10.1001/jamanetworkopen.2023.50680 PMC10799265

[4] Dunstan DW, Dogra S, Carter SE, Owen N. Sit less and move more for cardiovascular health: emerging insights and opportunities. Nat Rev Cardiol. 2021;18(9):637–48. Available from: https://www.nature.com/articles/s41569-021-00547-y. Cited 2024 Dec 4. 34017139 10.1038/s41569-021-00547-y

[5] Winkler EAH, Chastin S, Eakin EG, Owen N, Lamontagne AD, Moodie M, et al. Cardiometabolic impact of changing sitting, standing, and stepping in the workplace. Med Sci Sports Exerc. 2018;50(3):516–24. 29166319 10.1249/MSS.0000000000001453

[6] Owen N, Sugiyama T, Eakin EE, Gardiner PA, Tremblay MS, Sallis JF. Adults’ sedentary behavior determinants and interventions. Am J Prev Med. 2011;41(2):189–96. 21767727 10.1016/j.amepre.2011.05.013

[7] Neuhaus M, Healy GN, Dunstan DW, Owen N, Eakin EG. Workplace sitting and height-adjustable workstations: a randomized controlled trial. Am J Prev Med. 2014;46(1):30–40. 24355669 10.1016/j.amepre.2013.09.009

[8] McGuckin T, Sealey R, Barnett F. Planning for sedentary behaviour interventions: office workers’ survey and focus group responses. Perspect Public Health. 2017;137(6):316–21. 28345430 10.1177/1757913917698003

[9] Pereira MA, Mullane SL, Toledo MJL, Larouche ML, Rydell SA, Vuong B, et al. Efficacy of the ‘Stand and Move at Work’ multicomponent workplace intervention to reduce sedentary time and improve cardiometabolic risk: a group randomized clinical trial. Int J Behav Nutr Phys Act. 2020;17(1):1–11. 33109190 10.1186/s12966-020-01033-3 PMC7592578

[10] Healy GN, Winkler EAH, Eakin EG, Owen N, Lamontagne AD, Moodie M, et al. A cluster RCT to reduce workers’ sitting time: impact on cardiometabolic biomarkers. Med Sci Sports Exerc. 2017;49(10):2032–9. 28538025 10.1249/MSS.0000000000001328

[11] Healy GN, Eakin EG, Owen N, LaMontagne AD, Moodie M, Winkler EAH, et al. A cluster randomized controlled trial to reduce office workers’ sitting time: effect on activity outcomes. Med Sci Sports Exerc. 2016;48(9):1787–97. 27526175 10.1249/MSS.0000000000000972

[12] Edwardson CL, Yates T, Biddle SJH, Davies MJ, Dunstan DW, Esliger DW, et al. Effectiveness of the Stand More AT (SMArT) Work intervention: cluster randomised controlled trial. BMJ. 2018;10(363):3870. 10.1136/bmj.k3870 PMC6174726 30305278

[13] Zhu W, Gutierrez M, Toledo MJ, Mullane S, Stella AP, Diemar R, et al. Long-term effects of sit-stand workstations on workplace sitting: a natural experiment. J Sci Med Sport. 2018;21(8):811–6. Available from: https://pubmed.ncbi.nlm.nih.gov/29289496/. Cited 2023 Oct 10. 29289496 10.1016/j.jsams.2017.12.005

[14] Zhu W, Gutierrez M, Toledo MJ, Mullane S, Stella AP, Diemar R, et al. Long-term effects of sit-stand workstations on workplace sitting: a natural experiment. J Sci Med Sport. 2018;21(8):811–6. 29289496 10.1016/j.jsams.2017.12.005

[15] Commissaris DACM, Huysmans MA, Mathiassen SE, Srinivasan D, Koppes LLJ, Hendriksen IJM. Interventions to reduce sedentary behavior and increase physical activity during productive work: a systematic review. Scand J Work Environ Health. 2016;42(3):181–91. 26683116 10.5271/sjweh.3544

[16] Brierley ML, Chater AM, Smith LR, Bailey DP. The effectiveness of sedentary behaviour reduction workplace interventions on cardiometabolic risk markers: a systematic review. Sports Med. 2019;49(11):1739–67. 31429035 10.1007/s40279-019-01168-9

[17] Shrestha N, Kukkonen-Harjula KT, Verbeek JH, ljaz S, Hermans V, Pedisic Z. Workplace interventions for reducing sitting at work. Cochrane Database Syst Rev. 2018;6(6). 10.1002/14651858.CD010912.pub4 PMC6513236 29926475

[18] Chu AHY, Ng SHX, Tan CS, Win AM, Koh D, Müller-Riemenschneider F. A systematic review and meta-analysis of workplace intervention strategies to reduce sedentary time in white-collar workers. Obes Rev. 2016;17(5):467–81. 26990220 10.1111/obr.12388

[19] Buman MP, Mullane SL, Toledo MJ, Rydell SA, Gaesser GA, Crespo NC, et al. An intervention to reduce sitting and increase light-intensity physical activity at work: design and rationale of the “Stand & Move at Work” group randomized trial. Contemp Clin Trials. 2017;1(53):11–9. 10.1016/j.cct.2016.12.008 PMC5274555 27940181

[20] Mullane SL, Rydell SA, Larouche ML, Toledo MJL, Feltes LH, Vuong B, et al. enrollment strategies, barriers to participation, and reach of a workplace intervention targeting sedentary behavior. Am J Health Promot. 2019;33(2):225–36. 29986592 10.1177/0890117118784228 PMC7702267

[21] Buman MP, Mullane SL, Toledo MJ, Rydell SA, Gaesser GA, Crespo NC, et al. An intervention to reduce sitting and increase light-intensity physical activity at work: design and rationale of the ‘Stand & Move at Work’ group randomized trial. Contemp Clin Trials. 2017;53:11–9. Available from: https://pubmed-ncbi-nlm-nih-gov.ezproxy1.lib.asu.edu/27940181/. Cited 2021 Mar 31. 27940181 10.1016/j.cct.2016.12.008 PMC5274555

[22] Kozey-Keadle S, Libertine A, Lyden K, Staudenmayer J, Freedson PS. Validation of wearable monitors for assessing sedentary behavior. Med Sci Sports Exerc. 2011;43(8):1561–7. 21233777 10.1249/MSS.0b013e31820ce174

[23] Lyden K, Keadle SK, Staudenmayer J, Freedson PS. The activPALTM accurately classifies activity intensity categories in healthy adults. Med Sci Sports Exerc. 2017;49(5):1022–8. 28410327 10.1249/MSS.0000000000001177 PMC5469371

[24] Winkler EAH, Bodicoat DH, Healy GN, Bakrania K, Yates T, Owen N, et al. Identifying adults’ valid waking wear time by automated estimation in activPAL data collected with a 24 h wear protocol. Physiol Meas. 2016;37(10):1653–68. 27652827 10.1088/0967-3334/37/10/1653

[25] Abel M, Hannon J, Mullineaux D, Beighle A. Determination of step rate thresholds corresponding to physical activity intensity classifications in adults. J Phys Act Health. 2011;8(1):45–51. Available from: https://pubmed-ncbi-nlm-nih-gov.ezproxy1.lib.asu.edu/21297184/. Cited 2021 Dec 15. 21297184 10.1123/jpah.8.1.45

[26] Hillier TA, Rousseau A, Lange C, Lépinay P, Cailleau M, Novak M, et al. Practical way to assess metabolic syndrome using a continuous score obtained from principal components analysis. Diabetologia. 2006;49(7):1528–35. 16752171 10.1007/s00125-006-0266-8 PMC3505191

[27] Thomsen T, Aadahl M, Beyer N, Hetland ML, Løppenthin KB, Midtgaard J, et al. Sustained long-term efficacy of motivational counseling and text message reminders on daily sitting time in patients with rheumatoid arthritis: long-term follow-up of a randomized, parallel-group trial. Arthritis Care Res (Hoboken). 2020;72(11):1560–70. Available from: https://onlinelibrary.wiley.com/doi/full/10.1002/acr.24060. Cited 2023 Oct 10. 31507095 10.1002/acr.24060

[28] Laska MN, Lytle LA, Nanney MS, Moe SG, Linde JA, Hannan PJ. Results of a 2-year randomized, controlled obesity prevention trial: Effects on diet, activity and sleep behaviors in an at-risk young adult population. Prev Med (Baltim). 2016;89:230–6. Available from: https://pubmed.ncbi.nlm.nih.gov/27283096/. Cited 2023 Oct 10. 10.1016/j.ypmed.2016.06.001 PMC5038135 27283096

[29] Lioret S, Campbell KJ, Crawford D, Spence AC, Hesketh K, McNaughton SA. A parent focused child obesity prevention intervention improves some mother obesity risk behaviors: the Melbourne inFANT program. Int J Behav Nutr Phys Act. 2012;9. Available from: https://pubmed.ncbi.nlm.nih.gov/22925356/. Cited 2023 Oct 10. 10.1186/1479-5868-9-100 PMC3533722 22925356

[30] Lakerveld J, Bot SDM, Van der Ploeg HP, Nijpels G. The effects of a lifestyle intervention on leisure-time sedentary behaviors in adults at risk: the Hoorn prevention study, a randomized controlled trial. Prev Med (Baltim). 2013;57(4):351–6. Available from: https://pubmed.ncbi.nlm.nih.gov/23777672/. Cited 2023 Oct 10. 10.1016/j.ypmed.2013.06.011 23777672

[31] Thomsen T, Aadahl M, Beyer N, Hetland ML, Løppenthin KB, Midtgaard J, et al. Sustained long-term efficacy of motivational counseling and text message reminders on daily sitting time in patients with rheumatoid arthritis: long-term follow-up of a randomized, Parallel-Group Trial. Arthritis Care Res (Hoboken). 2020;72(11):1560–70. 31507095 10.1002/acr.24060

[32] Laska MN, Lytle LA, Nanney MS, Moe SG, Linde JA, Hannan PJ. Results of a 2-year randomized, controlled obesity prevention trial: Effects on diet, activity and sleep behaviors in an at-risk young adult population. Prev Med (Baltim). 2016;1(89):230–6. 10.1016/j.ypmed.2016.06.001 PMC5038135 27283096

[33] Lioret S, Campbell KJ, Crawford D, Spence AC, Hesketh K, McNaughton SA. A parent focused child obesity prevention intervention improves some mother obesity risk behaviors: the Melbourne inFANT program. Int J Behav Nutr Phys Act. 2012;28:9. 10.1186/1479-5868-9-100 PMC3533722 22925356

[34] Lakerveld J, Bot SDM, Van der Ploeg HP, Nijpels G. The effects of a lifestyle intervention on leisure-time sedentary behaviors in adults at risk: the Hoorn Prevention Study, a randomized controlled trial. Prev Med (Baltim). 2013;57(4):351–6. 10.1016/j.ypmed.2013.06.011 23777672

[35] Neuhaus M, Healy GN, Dunstan DW, Owen N, Eakin EG. Workplace sitting and height-adjustable workstations: a randomized controlled trial. Am J Prev Med. 2014;46(1):30–40. Available from: https://pubmed.ncbi.nlm.nih.gov/24355669/. Cited 2024 Dec 4. 24355669 10.1016/j.amepre.2013.09.009

[36] Peachey MM, Richardson JV, Tang A, Dal-Bello Haas V, Gravesande J. Environmental, behavioural and multicomponent interventions to reduce adults’ sitting time: a systematic review and meta-analysis. Br J Sports Med. 2020;54(6):315–25. Available from: https://pubmed.ncbi.nlm.nih.gov/30352864/. Cited 2024 Dec 4. 30352864 10.1136/bjsports-2017-098968

[37] Carr LJ, Swift M, Ferrer A, Benzo R. Cross-sectional examination of long-term access to sit-stand desks in a professional office setting. Am J Prev Med. 2016;50(1):96–100. 26437867 10.1016/j.amepre.2015.07.013

[38] Buckley JP, Mellor DD, Morris M, Joseph F. Standing-based office work shows encouraging signs of attenuating post-prandial glycaemic excursion. Occup Environ Med. 2014;71(2):109–11. 24297826 10.1136/oemed-2013-101823

[39] Dunstan DW, Kingwell BA, Larsen R, Healy GN, Cerin E, Hamilton MT, et al. Breaking up prolonged sitting reduces postprandial glucose and insulin responses. Diabetes Care. 2012;35(5):976–83. 22374636 10.2337/dc11-1931 PMC3329818

[40] Peddie MC, Bone JL, Rehrer NJ, Skeaff CM, Gray AR, Perry TL. Breaking prolonged sitting reduces postprandial glycemia in healthy, normal-weight adults: a randomized crossover trial. Am J Clin Nutr. 2013;98(2):358–66. 23803893 10.3945/ajcn.112.051763

[41] Latouche C, Jowett JBM, Carey AL, Bertovic DA, Owen N, Dunstan DW, et al. Effects of breaking up prolonged sitting on skeletal muscle gene expression. J Appl Physiol (1985). 2013;114(4):453–60. 23271697 10.1152/japplphysiol.00978.2012

[42] Bailey DP, Locke CD. Breaking up prolonged sitting with light-intensity walking improves postprandial glycemia, but breaking up sitting with standing does not. J Sci Med Sport. 2015;18(3):294–8. 24704421 10.1016/j.jsams.2014.03.008

[43] Crespo NC, Mullane SL, Zeigler ZS, Buman MP, Gaesser GA. Effects of standing and light-intensity walking and cycling on 24-h glucose. Med Sci Sports Exerc. 2016;48(12):2503–11. 27471786 10.1249/MSS.0000000000001062

[44] Kruse NT, Hughes WE, Benzo RM, Carr LJ, Casey DP. Workplace strategies to prevent sitting-induced endothelial dysfunction. Med Sci Sports Exerc. 2018;50(4):801–8. 29117072 10.1249/MSS.0000000000001484

[45] Padilla J, Fadel PJ. Prolonged sitting leg vasculopathy: contributing factors and clinical implications. Am J Physiol Heart Circ Physiol. 2017;313(4):722–8. 10.1152/ajpheart.00326.2017 PMC5668607 28733451

[46] Morishima T, Restaino RM, Walsh LK, Kanaley JA, Padilla J. Prior exercise and standing as strategies to circumvent sitting-induced leg endothelial dysfunction. Clin Sci. 2017;131(11):1045–53. 10.1042/CS20170031 PMC5516793 28385735

[47] Ekelund U, Steene-Johannessen J, Brown WJ, Fagerland MW, Owen N, Powell KE, et al. Does physical activity attenuate, or even eliminate, the detrimental association of sitting time with mortality? A harmonised meta-analysis of data from more than 1 million men and women. Lancet. 2016;388(10051):1302–10. 27475271 10.1016/S0140-6736(16)30370-1

[48] Ekelund U, Brown WJ, Steene-Johannessen J, Fagerland MW, Owen N, Powell KE, et al. Do the associations of sedentary behaviour with cardiovascular disease mortality and cancer mortality differ by physical activity level? A systematic review and harmonised meta-analysis of data from 850 060 participants. Br J Sports Med. 2019;53(14):886–94. 29991570 10.1136/bjsports-2017-098963

[49] Leonard KS, Mullane SL, Golden CA, Rydell SA, Mitchell NR, Koskan A, et al. Qualitative comparative analysis of the implementation fidelity of a workplace sedentary reduction intervention. BMC Public Health. 2022;22:1–11. 10.1186/s12889-022-13476-3. 35641923 10.1186/s12889-022-13476-3 PMC9158295

