# Correction: Maintenance effects of a multilevel workplace intervention to reduce sedentary time: twenty-four-month follow-up of the group randomized clinical trial ‘stand and move at work’

**DOI:** 10.1186/s12966-025-01770-3

**Published:** 2025-06-02

**Authors:** Krista S. Leonard, Miranda Larouche, Nathan R. Mitchell, Sarah A. Rydell, Meynard John Toledo, Sarah L. Mullane, Kristina Hasanaj, Junia N. de Brito, Matthew P. Buman, Mark A. Pereira

**Affiliations:** 1 College of Health Solutions, Arizona State University, Phoenix, USA; 2 School of Public Health, Department of Epidemiology & Community Health, University of Minnesota, Minneapolis, USA; 3 Center for Economic and Social Research, University of Southern California, Los Angeles, USA; 4 Johnson and Johnson Health and Wellness Solutions, New Brunswick, USA; 5 Feinberg School of Medicine, Northwestern University, Chicago, IL USA; 6 Medical School, Department of Family Medicine and Community Health, University of Minnesota, Minneapolis, MN USA

**Leonard et al. Int J Behav Nutr Phys Act (2025) 22:39.**

10.1186/s12966-025-01731-w.

After the publication of the original article, the authors reported that the name of an author, Junia N. de Brito, had been erroneously omitted. This correction presents the correct authorship.

The original article has been corrected.

