# Supplementary material for: Maintenance effects of a multilevel workplace intervention to reduce sedentary time: twenty-four-month follow-up of the group randomized clinical trial ‘Stand and Move at Work’
Source: Int J Behav Nutr Phys Act. 2025 Apr 7;22:39. doi: 10.1186/s12966-025-01731-w (PMC11978190; doi:10.1186/s12966-025-01731-w)
Supplement: Supplementary file 1 — Supplementary Material 1. [file 12966_2025_1731_MOESM1_ESM.docx]

| **Supplementary Table 1.** Comparison of demographic, activity, and cardiometabolic risk biomarkers of the 0-, 12-, and 24-month samples by study arm at baseline. | | | | | | | | | | | | | | | | | | | | | |
| --- | --- | --- | --- | --- | --- | --- | --- | --- | --- | --- | --- | --- | --- | --- | --- | --- | --- | --- | --- | --- | --- |
|  |  |  | *MOVE+* | | | | | | | | |  | *STAND+* | | | | | | | | |
|  |  |  | Baseline sample | |  | 12-month analytical sample | |  | 24-month analytical sample | |  |  | Baseline sample | |  | 12-month analytical sample | |  | 24-month analytical sample | |  |
|  |  |  | n | (%) |  | n | (%) |  | n | (%) |  |  | n | (%) |  | n | (%) |  | n | (%) |  |
| N worksites |  |  | 12 | (50.0) |  | 12 | (50.0) |  | 12 | (50.0) |  |  | 12 | (50.0) |  | 12 | (50.0) |  | 12 | (50.0) |  |
| N individuals |  |  | 276 | (43.8) |  | 247 | (45.8) |  | 216 | (46.6) |  |  | 354 | (56.2) |  | 292 | (54.2) |  | 248 | (53.4) |  |
| Region | | | | | | | | | | | | | | | | | | | | |  |
|  |  | Phoenix, Arizona | 138 | (50.0) |  | 116 | (47.5) |  | 106 | (49.1) |  |  | 194 | (54.8) |  | 157 | (53.8) |  | 137 | (55.2) |  |
|  |  | Minneapolis/St. Paul, Minnesota | 138 | (50.0) |  | 128 | (52.5) |  | 110 | (50.9) |  |  | 160 | (45.2) |  | 135 | (46.2) |  | 111 | (44.8) |  |
| Age, M±SD (years) | |  | 43.3 | ±10.8 |  | 43.5 | ±10.5 |  | 44.7±10.4 | |  |  | 45.6 | ±11.4 |  | 46.4±10.9 | |  | 46.9±10.7 | |  |
| Race |  |  |  |  |  |  |  |  |  |  |  |  |  |  |  |  |  |  |  |  |  |
|  |  | Non-Hispanic White | 205.0 | (74.3) |  | 180.0 | (73.8) |  | 158.0 | (73.1) |  |  | 239.0 | (67.5) |  | 201.0 | (68.8) |  | 168.0 | (67.7) |  |
|  |  | Hispanic | 30.0 | (10.9) |  | 28.0 | (11.5) |  | 25.0 | (11.6) |  |  | 57.0 | (16.1) |  | 42.0 | (14.4) |  | 35.0 | (14.1) |  |
|  |  | Non-Hispanic Black | 6.0 | (2.2) |  | 6.0 | (2.5) |  | 4.0 | (1.9) |  |  | 20.0 | (5.7) |  | 18.0 | (6.2) |  | 18.0 | (7.3) |  |
|  |  | Non-Hispanic Asian | 17.0 | (6.2) |  | 15.0 | (6.1) |  | 15.0 | (6.9) |  |  | 15.0 | (4.2) |  | 12.0 | (4.1) |  | 10.0 | (4.0) |  |
|  |  | Other/Multiracial/Unknown | 18.0 | (6.5) |  | 15.0 | (6.1) |  | 14.0 | (6.5) |  |  | 23.0 | (6.5) |  | 19.0 | (6.5) |  | 17.0 | (6.9) |  |
| Female |  |  | 173.0 | (62.7) |  | 151.0 | (61.9) |  | 133.0 | (61.6) |  |  | 296.0 | (83.6) |  | 243.0 | (83.2) |  | 206.0 | (83.1) |  |
| Education |  |  |  |  |  |  |  |  |  |  |  |  |  |  |  |  |  |  |  |  |  |
|  |  | Less than college | 15.0 | (5.4) |  | 14.0 | (5.7) |  | 14.0 | (6.5) |  |  | 14.0 | (4.0) |  | 10.0 | (3.4) |  | 8.0 | (3.2) |  |
|  |  | College/Some college | 151.0 | (54.7) |  | 134.0 | (54.9) |  | 120.0 | (55.6) |  |  | 234.0 | (66.1) |  | 196.0 | (67.1) |  | 169.0 | (68.1) |  |
|  |  | Graduate/Professional | 98.0 | (35.5) |  | 87.0 | (35.7) |  | 75.0 | (34.7) |  |  | 93.0 | (26.3) |  | 76.0 | (26.0) |  | 62.0 | (25.0) |  |
|  |  | Unknown | 12.0 | (4.4) |  | 9.0 | (3.7) |  | 7.0 | (3.2) |  |  | 13.0 | (3.7) |  | 10.0 | (3.4) |  | 9.0 | (3.6) |  |
| Work sector |  |  |  |  |  |  |  |  |  |  |  |  |  |  |  |  |  |  |  |  |  |
|  |  | Academic | 94.0 | (34.1) |  | 83.0 | (34.0) |  | 70.0 | (32.4) |  |  | 119.0 | (33.6) |  | 100.0 | (34.2) |  | 86.0 | (34.7) |  |
|  |  | Industry/healthcare | 83.0 | (30.1) |  | 72.0 | (29.5) |  | 64.0 | (29.6) |  |  | 123.0 | (34.8) |  | 95.0 | (32.5) |  | 74.0 | (29.8) |  |
|  |  | Government | 99.0 | (35.9) |  | 89.0 | (36.5) |  | 82.0 | (38.0) |  |  | 112.0 | (31.6) |  | 97.0 | (33.2) |  | 88.0 | (35.5) |  |
| Job type |  |  |  |  |  |  |  |  |  |  |  |  |  |  |  |  |  |  |  |  |  |
|  |  | Executive | 39.0 | (14.1) |  | 36.0 | (14.8) |  | 32.0 | (14.8) |  |  | 43.0 | (12.2) |  | 40.0 | (13.7) |  | 31.0 | (12.5) |  |
|  |  | Professional | 155.0 | (56.2) |  | 139.0 | (57.0) |  | 124.0 | (57.4) |  |  | 182.0 | (51.4) |  | 152.0 | (52.1) |  | 130.0 | (52.4) |  |
|  |  | Clerical | 72.0 | (26.1) |  | 62.0 | (25.4) |  | 54.0 | (25.0) |  |  | 120.0 | (33.9) |  | 94.0 | (32.2) |  | 82.0 | (33.1) |  |
|  |  | Unknown | 10.0 | (3.6) |  | 7.0 | (2.9) |  | 6.0 | (2.8) |  |  | 9.0 | (2.5) |  | 6.0 | (2.1) |  | 5.0 | (2.0) |  |
| Baseline behavioral outcomes, *M±SD* | | | | | | | | | | | | | | | | | | | | |  |
|  | Work periods (min per 8 h workday) | |  | |  |  | |  |  | |  |  |  | |  |  | |  |  | |  |
|  |  | Sitting | 337.9 | ±73.1 |  | 335.0 | ±74.9 |  | 330.9 | ±76.9 |  |  | 330.8 | ±79.8 |  | 330.6 | ±79.2 |  | 329.4 | (81.4) |  |
|  |  | Standing | 104.3 | ±69.8 |  | 107.0 | ±72.0 |  | 111.1 | ±74.6 |  |  | 113.6 | ±75.9 |  | 113.4 | ±74.8 |  | 114.1 | (76.8) |  |
|  |  | LPA | 31.4 | ±15.4 |  | 31.4 | ±15.6 |  | 31.3 | ±15.6 |  |  | 29.9 | ±14.0 |  | 30.3 | ±14.5 |  | 30.7 | (15.2) |  |
|  |  | MVPA | 6.4 | ±5.3 |  | 6.5 | ±5.5 |  | 6.7 | ±5.6 |  |  | 5.7 | ±4.7 |  | 5.7 | ±4.8 |  | 5.7 | (4.6) |  |
|  |  | LPA + MVPA | 37.8 | ±17.2 |  | 38.0 | ±17.4 |  | 38.0 | ±17.2 |  |  | 35.6 | ±15.4 |  | 36.0 | ±16.0 |  | 36.5 | (16.7) |  |
|  |  | Prolonged sitting (>30 min) | 161.8 | ±85.7 |  | 158.6 | ±86.1 |  | 153.2 | ±84.3 |  |  | 142.3 | ±90.5 |  | 142.6 | ±90.4 |  | 141.5 | (92.3) |  |
|  |  | Sit-stand transitions (n/sitting-hr) | 6.1 | ±6.6 |  | 6.0 | ±3.7 |  | 6.2 | ±3.8 |  |  | 7.8 | ±5.8 |  | 7.0 | ±4.2 |  | 7.0 | (4.3) |  |
|  | Total time (min per day) | |  |  |  |  |  |  |  |  |  |  |  |  |  |  | |  |  | |  |
|  |  | Sitting | 624.9 | ±87.4 |  | 621.0 | ±89.9 |  | 616.0 | ±91.3 |  |  | 619.8 | ±95.0 |  | 619.1 | ±96.3 |  | 616.6 | ±100.7 |  |
|  |  | Standing | 236.4 | ±75.4 |  | 239.6 | ±77.8 |  | 244.0 | ±79.2 |  |  | 243.1 | ±82.9 |  | 242.7 | ±83.7 |  | 244.6 | ±87.8 |  |
|  |  | LPA | 80.4 | ±28.3 |  | 80.9 | ±29.0 |  | 81.3 | ±30.0 |  |  | 79.5 | ±27.7 |  | 80.6 | ±28.3 |  | 81.3 | ±28.7 |  |
|  |  | MVPA | 18.2 | ±7.2 |  | 18.5 | ±7.3 |  | 18.7 | ±7.3 |  |  | 17.7 | ±7.6 |  | 17.6 | ±7.8 |  | 17.5 | ±7.8 |  |
|  |  | LPA + MVPA | 98.6 | ±31.9 |  | 99.4 | ±32.7 |  | 100.0 | ±33.6 |  |  | 97.1 | ±31.4 |  | 98.2 | ±32.1 |  | 98.8 | ±32.7 |  |
|  |  | Prolonged sitting (>30 min) | 322.5 | ±103.0 |  | 318.4 | ±102.9 |  | 314.6 | ±104.0 |  |  | 308.3 | ±107.2 |  | 308.8 | ±108.6 |  | 306.9 | ±109.8 |  |
|  |  | Sit-stand transitions (n/sitting-hr) | 5.4 | ±1.9 |  | 5.6 | ±1.8 |  | 5.6 | ±1.9 |  |  | 6.0 | ±2.2 |  | 5.9 | ±2.0 |  | 5.9 | ±2.1 |  |
| Baseline cardiometabolic risk biomarkers M±SD | | | | | | | | | | | | | | | | | | | | |  |
|  | Total sample | |  |  |  |  | |  |  |  |  |  |  |  |  |  |  |  |  |  |  |
|  |  | CMR (sum of Z scores) | 0.0 | ±0.6 |  | 0.0 | ±0.6 |  | 0.0 | ±0.6 |  |  | 0.0 | ±0.7 |  | 0.0 | ±0.7 |  | 0.0 | ±0.7 |  |
|  |  | Fasting glucose (mg/dL) | 91.0 | ±14.1 |  | 91.4 | ±14.0 |  | 91.9 | ±14.5 |  |  | 96.5 | ±37.1 |  | 95.7 | ±35.9 |  | 96.2 | ±37.3 |  |
|  |  | Fasting insulin (uU/mL) | 71.1 | ±55.0 |  | 70.9 | ±50.0 |  | 72.8 | ±52.0 |  |  | 77.6 | ±57.3 |  | 76.7 | ±58.0 |  | 75.5 | ±55.0 |  |
|  |  | HDL-cholesterol (mg/dL) | 56.4 | ±16.5 |  | 56.2 | ±16.7 |  | 56.5 | ±16.4 |  |  | 60.1 | ±18.8 |  | 59.8 | ±17.7 |  | 60.1 | ±17.4 |  |
|  |  | Triglycerides (mg/dL) | 120.3 | ±68.1 |  | 123.4 | ±69.9 |  | 126.9 | ±71.4 |  |  | 121.0 | ±74.4 |  | 118.1 | ±63.1 |  | 116.2 | ±62.5 |  |
|  |  | Diastolic BP (mm Hg) | 77.4 | ±10.7 |  | 77.9 | ±10.8 |  | 78.4 | ±10.9 |  |  | 77.7 | ±10.7 |  | 78.0 | ±10.5 |  | 78.2 | ±10.5 |  |
|  |  | Systolic BP (mm Hg) | 123.4 | ±15.7 |  | 124.0 | ±15.9 |  | 124.8 | ±16.1 |  |  | 124.9 | ±16.4 |  | 125.5 | ±15.6 |  | 126.1 | ±15.6 |  |
|  |  | LDL-cholesterol (mg/dL) | 113.1 | ±34.1 |  | 113.9 | ±34.2 |  | 113.2 | ±31.5 |  |  | 110.8 | ±30.3 |  | 111.5 | ±30.1 |  | 110.1 | ±30.1 |  |
|  |  | Weight (kg) | 82.9 | ±20.8 |  | 83.7 | ±20.3 |  | 84.1 | ±20.8 |  |  | 82.2 | ±22.8 |  | 82.9 | ±22.2 |  | 83.5 | ±22.1 |  |
|  |  | BMI (kg/m2) | 28.7 | ±6.4 |  | 28.9 | ±6.4 |  | 29.1 | ±6.5 |  |  | 29.8 | ±7.6 |  | 30.0 | ±7.5 |  | 30.2 | ±7.6 |  |
|  |  | Total body fat (%) | 32.8 | ±10.2 |  | 33.0 | ±10.4 |  | 33.2 | ±10.4 |  |  | 36.3 | ±9.6 |  | 36.7 | ±9.6 |  | 36.9 | ±9.7 |  |
| Note. CMR = summary continuous metabolic risk score; HDL = high-density lipoprotein; BP = blood pressure; BMI = body mass index. 12-month analytical sample included three individuals with missing demographic data. | | | | | | | | | | | | | | | | | | | | | |
